# Supplementary material for: Cyclic Game Dynamics Driven by Iterated Reasoning
Source: PLoS One. 2013 Feb 18;8(2):e56416. doi: 10.1371/journal.pone.0056416 (PMC3575333; doi:10.1371/journal.pone.0056416)

Figure S2

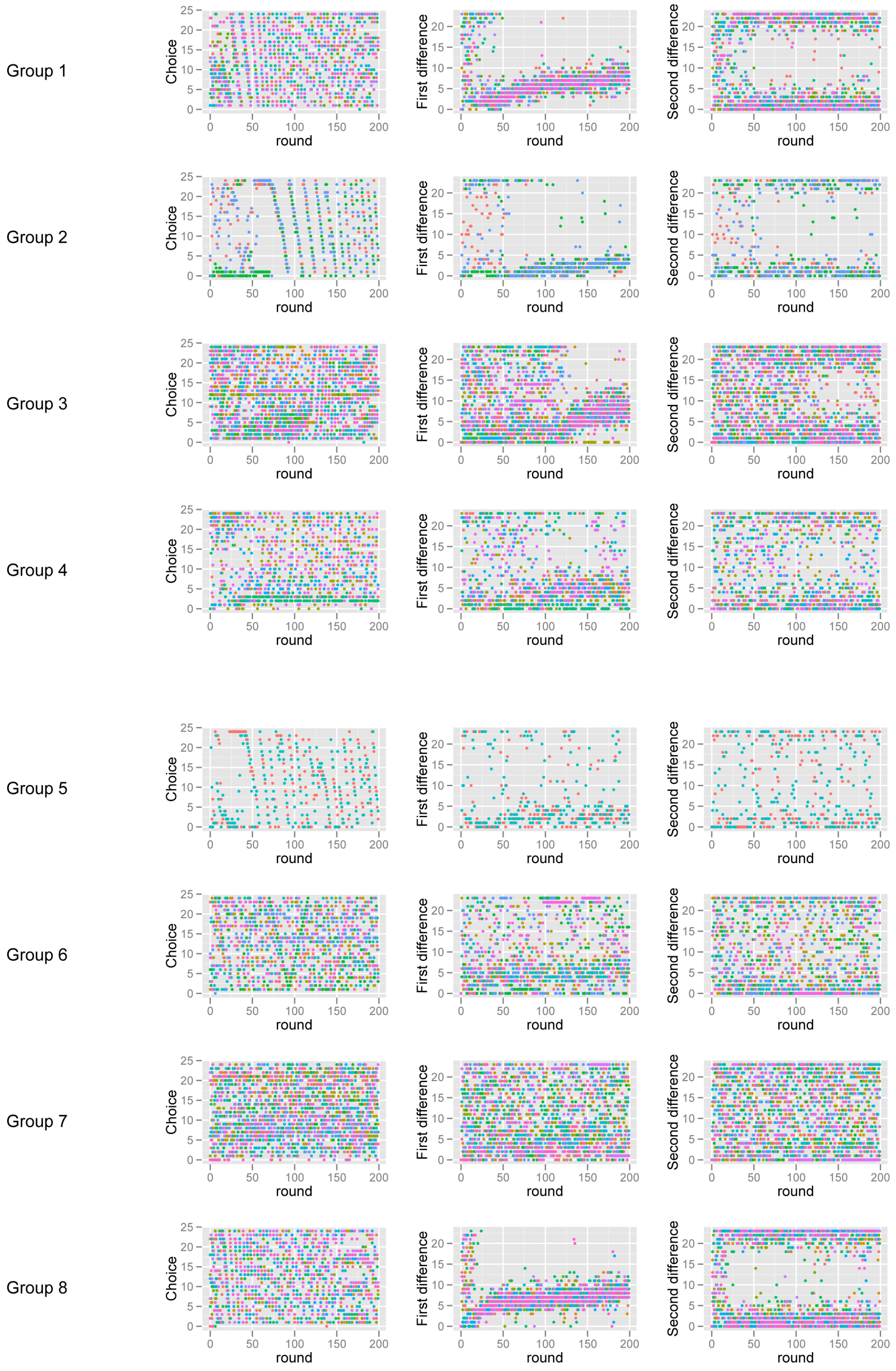
Group 1
Group 2
Group 3
Group 4
Group 5
Group 6
Group 7
Group 8
Choice
First difference
Second difference
round

Group 9

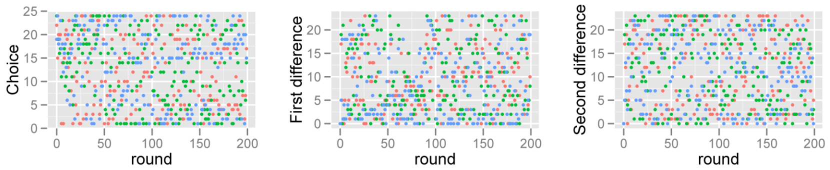

Group 10

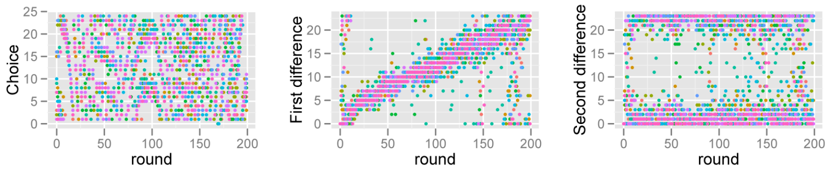

Group 11

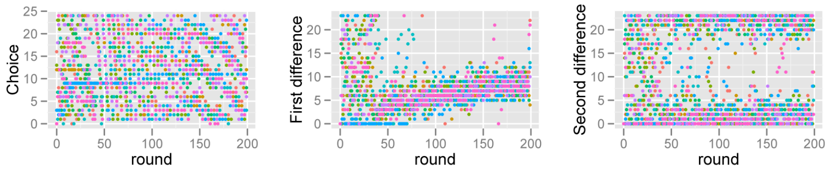

Group 12

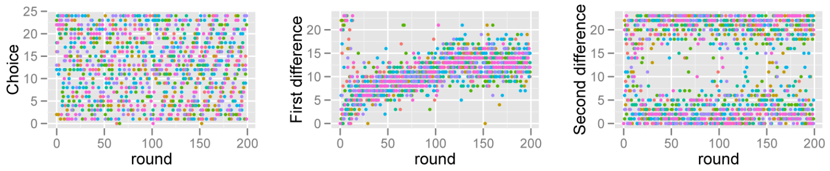

Group 13

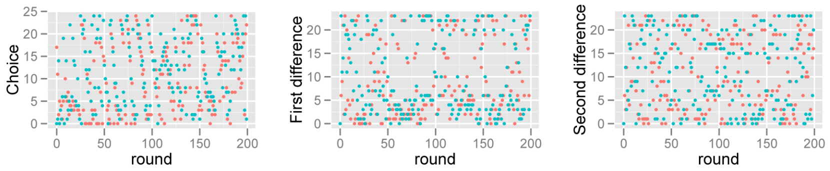

Group 14

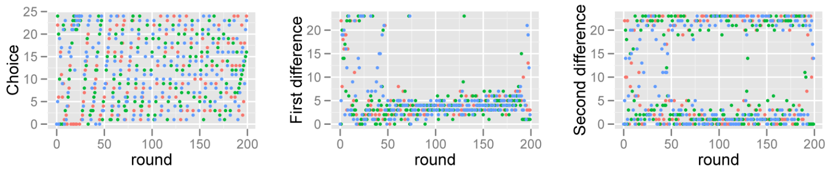

Group 15

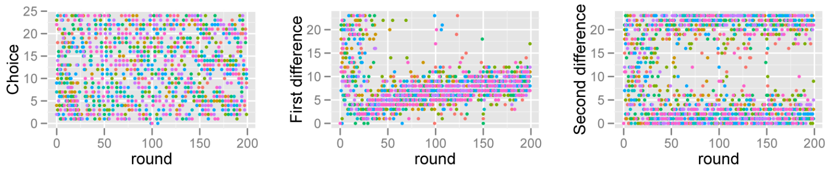

Group 16

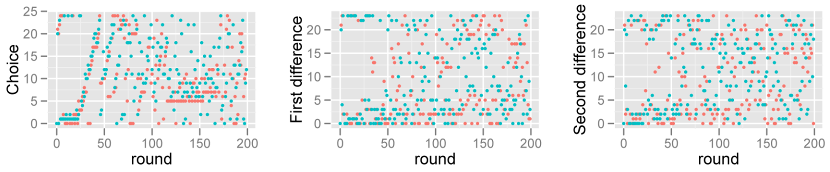

Group 17

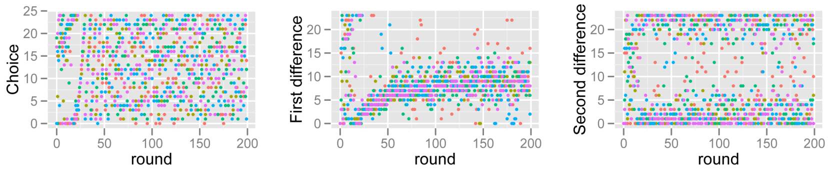

Group 18

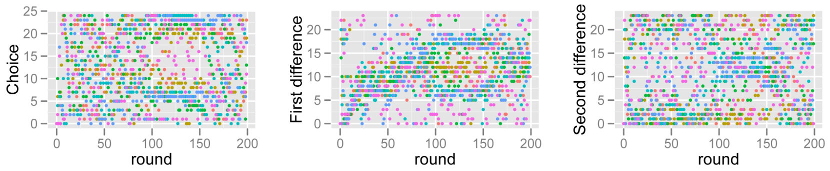

Group 19

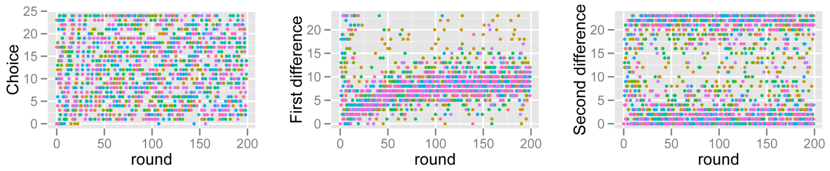

Group 20

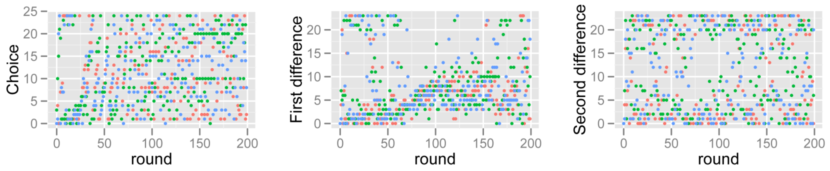

Group 21

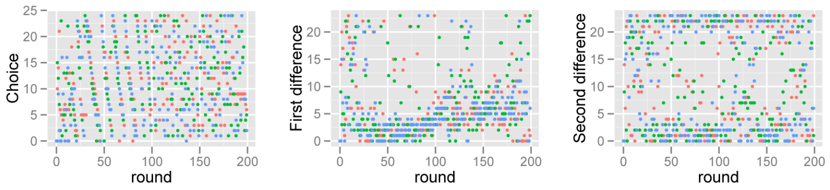

Group 22

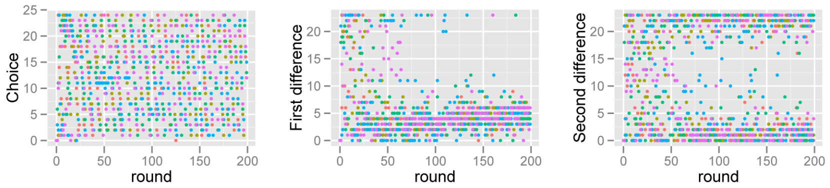

Discard 1

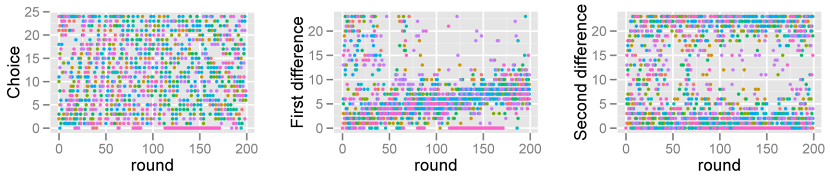

Discard 2

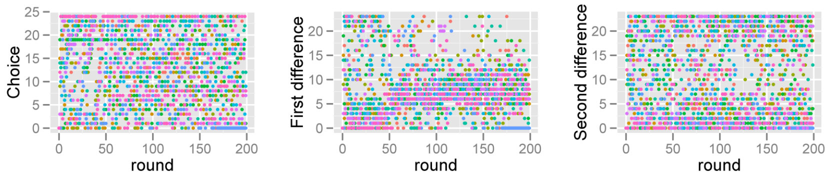

Discard 3

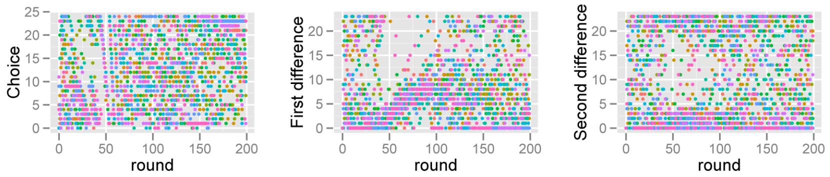

Discard 4

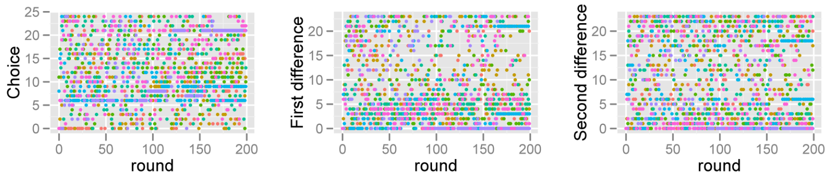

Discard 5

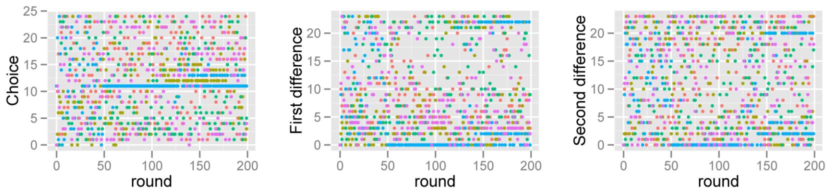

Discard 6

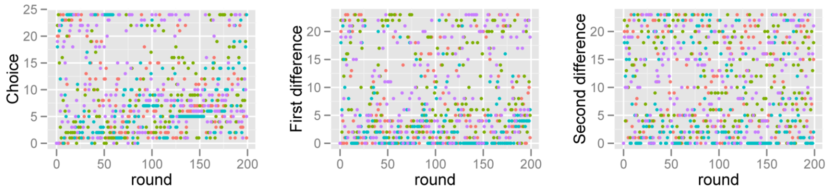

Discard 7

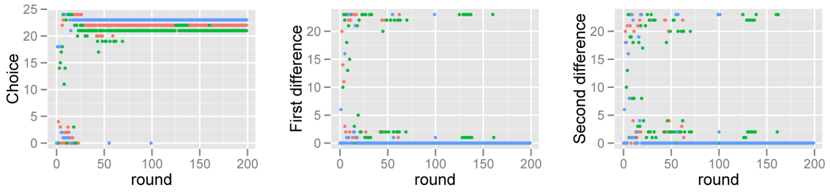

Supplement: Figure S1 — Choice, rate, and acceleration plots for groups 1–22, and for 7 groups excluded from analysis. Each row in this figure gives three representations of the raw data for one group. The three columns plot choice, rate, and then acceleration against time. Dot colors distinguish group members. While choice data (in the first column) seems disordered, rate and acceleration reveal group-level patterns. These plots give a sense of both the similarities and heterogeneity between groups. (PDF) [file pone.0056416.s001.pdf]
